# PAR2 Promoter Hypomethylation Regulates PAR2 Gene Expression and Promotes Lung Adenocarcinoma Cell Progression

**DOI:** 10.1155/2021/5542485

**Published:** 2021-04-15

**Authors:** Kuan Wu, Lei Xu, Ling Cheng

**Affiliations:** ^1^Department of Tumor Radiotherapy, First People's Hospital of Fuyang, 311400 Hangzhou, China; ^2^Department of General Medicine, First People's Hospital of Fuyang, 311400 Hangzhou, China; ^3^Shanghai Engineering Research Center of Pharmaceutical Translation, 200000 Shanghai, China

## Abstract

**Objective:**

Protease-activated receptor-2 (PAR2) also known as F2RL1 is a G protein-coupled receptor that intimately correlates with cancer occurrence. DNA methylation turns out a vital mechanism regulating gene expression, while PAR2 promoter methylation is proven to be involved in cancer development. Hence, this study attempted to clarify the molecular mechanism by which PAR2 mediates lung adenocarcinoma (LUAD) progression, via identifying the effect of PAR2 promoter methylation on LUAD cell progression.

**Methods:**

Associations of PAR2 promoter methylation with PAR2 gene expression and prognosis of LUAD patients were analyzed via bioinformatics analysis. PAR2 promoter methylation and gene expression at the cellular level were measured using methylation-specific PCR, qRT-PCR, and Western blot assays. DNA methyltransferase inhibitor 5-AzadC was used to treat cells to assess PAR2 gene expression alteration. Cell biological behaviors upon PAR2 overexpression were characterized via MTT, wound healing assay, and Transwell assay.

**Results:**

Bioinformatics analysis revealed that PAR2 promoter methylation was negatively related to PAR2 gene expression, while PAR2 promoter hypermethylation and low gene expression indicated favorable LUAD prognosis. Besides, it turned out that PAR2 presented upregulated expression and hypomethylated promoter in LUAD cells. Moreover, PAR2 gene expression was elevated in cells treated with 5-AzadC, and the proliferative, migratory, and invasive capabilities of cells with 5-AzadC or high PAR2 gene expression were all enhanced.

**Conclusion:**

In sum, PAR2 promoter hypomethylation potentiates LUAD cell progression, in turn affecting the prognosis of LUAD patients.

## 1. Introduction

Lung cancer is one of the most prevalent cancers and the most crucial cause for cancer death worldwide [1], while high metastasis is considered the predominant factor resulting in relatively high mortality of lung cancer. A study suggested that the proteases secreted by cancer cells and adjacent noncancer cells act a vital part in tumor metastasis [2]. Ubiquitin-specific protease 7, for example, is regarded as capable of inducing metastasis of osteosarcoma, and another membrane serine protease ST14 is believed to be involved in the occurrence of breast cancer [3, 4]. Additionally, there is evidence presenting that kallikrein-related peptidase 6 (KLK6) and trypsin, two members of the serine protease family, can activate protease-activated receptor-2 (PAR2) to boost the growth of lung adenocarcinoma (LUAD) cells, thus to advance cancer progression [5–7]. Given the abovementioned research, we reasoned that PAR2 as an important receptor of serine protease also participates in regulating lung cancer development.

PAR2, a G protein-coupled receptor [8], is believed to be implicated in microtubule regulation, cell growth, inflammation, pathological insults, and other physiological processes [9, 10]. In various cancers, PAR2 also shows values in tumorigenesis. As reported, PAR2 expression in ovarian cancer, cervical cancer, and lung cancer is significantly upregulated [11–13], which contributes to accelerated cancer progression via increasing tumor cell proliferation, migration, and invasion [13–16]. As for LUAD, there also exists a linkage with PAR2. A study found that by means of regulating epidermal growth factor receptor- (EGFR-) related signaling, PAR2 can induce the proliferation of LUAD cells A549, and it was also revealed that PAR2 can enhance cell migratory ability by suppressing miR-125b^16^. Nevertheless, despite the oncogenic role of PAR2 that is identified in several studies, the specific molecular mechanism in LUAD requires to be further discussed.

Enormous studies validated the presence of gene exogeneity alteration like CpG island methylation in human cancers [17, 18]. In lung cancer, DNA methylation occurs in tumor-related genes in early clinical diagnosis and is observed to be gradually enhanced with disease progression. In addition, a large number of methylated genes in cancer tissue are discovered to be intimately implicated in the occurrence of lung cancer. Hence, a study regarding the correlation between PAR2 promoter methylation and LUAD cell growth and mobility is of vital significance.

In the present research, we applied bioinformatics analysis and found that in LUAD cells, PAR2 promoter methylation presented a reverse correlation with PAR2 gene expression and is also related to the prognosis of LUAD patients. Hence, we reasoned that PAR2 promoter methylation level may regulate the proliferation, migration, invasion, and other cell malignant behaviors of LUAD via affecting PAR2 gene expression level. In the future, PAR2 may become a biomarker used for indicating LUAD malignant progression, which is of vital significance for LUAD diagnosis and treatment.

## 2. Materials and Methods

### 2.1. Bioinformatics Analysis

Relevant methylation (tumor: *n* = 475; normal: *n* = 32) and mRNA files (tumor: *n* = 535; normal: *n* = 59) accessed from TCGA-LUAD dataset were taken for bioinformatics analysis. The methylation sequencing platform is illumina human methylation 450. All profiles were then converted to expression data into matrixes. R package “limma” was utilized for data normalization, and the Wilcoxon rank-sum test was employed to screen genes with differential promoter methylation or gene expression. The package “MethylMix” was sequentially applied for identifying candidate methylation-driven genes which met the criteria of ∣logFC | >0.2, adjust *p* < 0.05, and Cor < −0.3. Correlation analysis for the promoter methylation/gene expression of the methylation-driven gene of interest and the prognosis of LUAD patients was performed using the “survival” package.

### 2.2. Cell Culture

Normal control human bronchial epithelial cell line BEAS-2B (BNCC100240) and experimental human LUAD cell lines H1299 (BNCC100268) and A549 (BNCC100215), as instructed, were grown in Roswell Park Memorial Institute-1640 (RPMI-1640) medium. The medium was added with 10% fetal bovine serum (FBS) (Gibco, Grand Island, NY, USA), along with 100 U/ml penicillin/streptomycin (Corning, NY, USA). All cell lines were ordered from BeNa Culture Collection (BNCC). Culture environment was set with 5% CO_2_ and a temperature of 37°C.

### 2.3. Cell Transfection

PAR2-overexpressed vector (oe-PAR2) (100 nmol/L) and its negative control (oe-NC) (100 nmol/L) were ordered from GenePharma (Shanghai, China). Before transfection, an estimate of 1 × 10^5^ cells were grown in a 12-well plate. Then, oe-PAR2 or oe-NC was taken for cell transfection using the LipoFiter assay kit (Hanbio, Shanghai, China), per the manufacturer's instructions. 48 h posttransfection, total RNA, and proteins were isolated for further experiments.

### 2.4. Quantitative Real-Time PCR (qRT-PCR)

qRT-PCR was implemented as instructed by Balal's study [19]. As recommended, total RNA extracted from cells with the QIAsymphony RNA Kit (Qiagen, Germany) was denatured via a water-bath at 65°C for 15 min and determined for concentration with NanoDrop2000. Then, 2 *μ*g of total RNA was taken and reversely transcribed into complementary DNA (cDNA) with the QuantiTect Reverse Transcription Kit (Qiagen, Germany) and Oligo dT primers. The specific process was as below: 16°C for 30 min, 42°C for 30 min, and 85°C for 5 min. After that, the ABI 7900HT instrument (Applied Biosystems, USA) was run to perform qRT-PCR with a starting temperature of 95°C lasting 5 min, followed by 40 cycles of cDNA denaturation (95°C for 15 s), and annealing/extension (60°C for 1 min). Sequencing was carried out with the QuantiFast SYBR® Green PCR Kit (Qiagen, Germany). With the tubulin as the internal reference, relative mRNA expression was examined by 2^-*ΔΔ*Ct^. Three independent experiments were conducted, and the primer sequences are listed in Supplementary Table 1.

### 2.5. Western Blot

Following diverse treatments, cells of each experimental group were harvested and washed with PBS. Protein samples obtained from 10 min of cell lysis using RIPA buffer were taken to be centrifuged, and the supernatant was taken for protein quantification with the BCA protein assay kit (Thermo Fisher Scientific, USA). After that, proper samples were subjected to a 10-min water-bath at 95°C together with loading buffer (10 *μ*l) and, then, separated by SDS-PAGE. After electrophoresis, under a voltage of 100 A, the proteins obtained were shifted to a nitrocellulose membrane within 120 min. Blocking solution 5% BSA/TBST was added, followed by addition of primary antibodies overnight at 4°C. Before and after addition of secondary antibody goat anti-rabbit IgG coupled with horseradish peroxidase at room temperature, the membrane was washed by 1 × TBST three times. The ECL kit from Solarbio (Beijing, China) was run to perform luminous reaction. At last, an automated Western blot system was applied to measure protein expression. Antibodies used here are detailed in Supplementary Table 2. Three repetitions were performed for this experiment.

### 2.6. Methylation-Specific PCR (MSP)

Promoter methylation was examined by means of MSP. In short, a DNA isolation kit was used to extract total DNA from cells, and the MethylDetectorTM bisulfite modification kit (Active Motif) was employed to perform sulfite modification. All unmethylated cytosine residues in DNA were converted into uracil, with no influence posed on methylated cytosine. The above procedures were carried out in accordance with the standard process. Subsequentially, with the DNA modified by 1 ng of sulfite as the template, MSP (2 × Taq PCR MasterMix with Dye, Solarbio) was conducted (annealing temperature: 60°C for methylation and 58°C for unmethylation), followed by an electrophoresis with 2% agarose gel. PCR results were analyzed on a Gel Imager and representative of three independent experiments. See Supplementary Table 1 for specific primer sequences.

### 2.7. MTT Assay

Cells at 1 × 10^4^ cells/ml (200 *μ*l) were precultured in a 96-well plate, with three repeated wells set for each treatment. At indicated time points (24, 48, 72, 96, and 120 h), sterile MTT reagent (Beyotime, Shanghai, China) was added into each well to test cell proliferation in accordance with the standard process. Optical density (OD) values at 570nm nm were read on the enzyme-labeled instrument (Molecular Devices, Sunnyvale, CA, USA).

### 2.8. Wound Healing Assay and Transwell Invasion Assay

Wound healing assay: cells (5 × 10 [6]) grown to 80% in confluence in a 6-well plate were wounded on the single layer across the well center with a pipette tip (200 *μ*l). Then, the cells were continuously cultured for further 48 h with fresh medium. An inverted microscope was used to observe would healing conditions at 0 and 48 h, respectively.

Transwell assay: a 24-well plate with Transwell inserts (8 *μ*m in aperture; BD Biosciences) was applied to test cell invasive capability. At first, cells were cultured for 24 h and then digested into single-cell suspension. Then, 1 ml of cell suspension was taken for a centrifugation at 1500 g lasting 5 min, with the supernatant discarded. Following that, the cells were suspended by 200 *μ*l of serum-free medium and planted into the upper chamber which was coated with Matrigel matrix-coated upper chambers before. 10% FBS-supplemented DMEM was added to the lower chamber to stimulate cell invasion. After 48 h, 4% paraformaldehyde and 0.5% crystal violet were used for cell fixation and staining. Cells that did not enter the lower chamber were gently removed with a cotton swab. Four views under an inverted microscope were selected at random to count cells that invaded.

### 2.9. Statistical Analysis

Data for statistical analysis on GraphPad Prism 6.0 (LaJolla, CA) were from three independent experiments and presented as mean ± standard deviation. For comparison of two sets of data, student's *t*-test was allowed in the case of two groups, while one-way analysis of variance (ANOVA) was applied upon more than two groups. Statistically significant difference was defined when *p* < 0.05.

## 3. Results

### 3.1. PAR2 Promoter Methylation Is Predicted to Be Associated with PAR2 Gene Expression Level and the Prognosis of LUAD Patients via Bioinformatics Analysis

**Figure 1:**
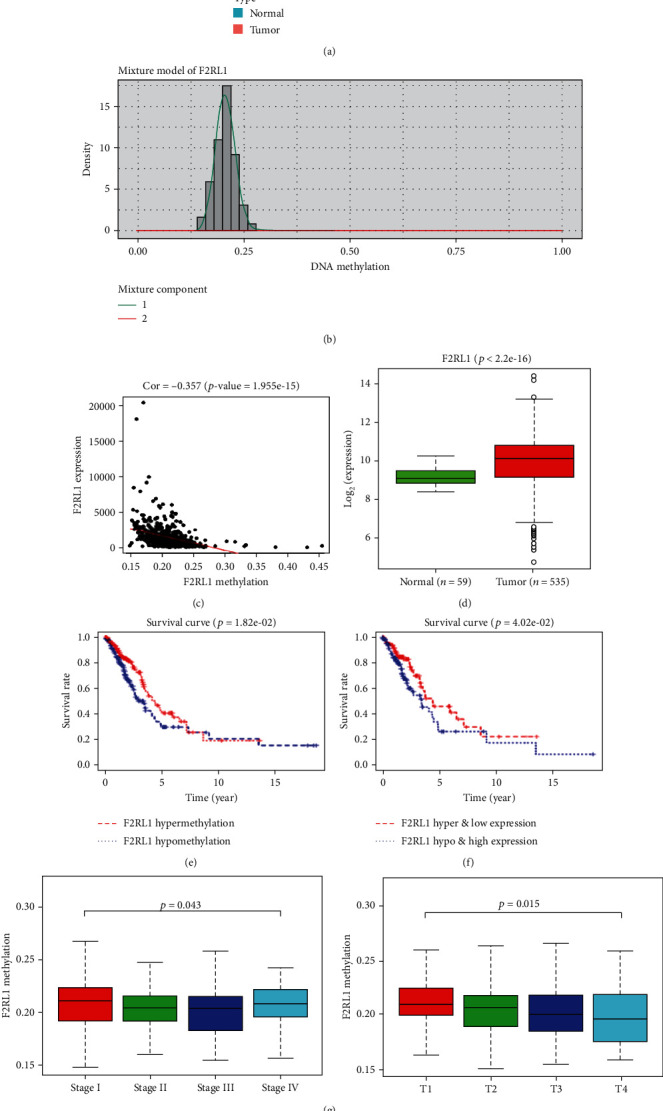
Bioinformatics analysis suggests that PAR2 promoter methylation is related to PAR2 mRNA expression and the prognosis of LUAD patients. (a) “MethylMix” package was used to screen methylation-driven genes from TCGA-LUAD dataset as plotted in a heat map, with red representing high methylation and green representing low methylation. PAR2 was identified and its (b) promoter methylation level was detected in tumor samples (histogram) and normal tissue samples (horizontal black line) in TCGA-LUAD dataset. (c) Correlation analysis for PAR2 promoter methylation and gene expression. (d) Relative expression level of PAR2 in TCGA was examined. (e) Survival analysis shows overall survival difference between high/low PAR2 promoter methylation level in LUAD patients (red line: hypermethylation; blue line: hypomethylation). (f) Overall survival difference in LUAD patients between hyper- and low-expression (red line) and hypo- and high-expression (blue line) groups. Afterwards, (g) the promoter methylation level of PAR2 was determined in different tumor stages and T stages combined with clinical information. (h) Correlation analysis between methylation level of three PAR2-related methylated sites and PAR2 expression level, respectively.

Totally, 74 methylation-driven genes were screened out from TCGA-LUAD dataset using the “MethylMix” package (Figure 1(a)). Of them, PAR2 (also known as F2RL1) was observed to have significantly poor promoter methylation (Figure 1(b)) but highly expressed PAR2 mRNA (Figure 1(d)) in cancer tissue. Correlation analysis noted that there existed a reverse correlation between promoter methylation and mRNA expression of PAR2 (Figure 1(c)). Published literature reported that PAR2 promoter hypomethylation is associated with tumor cell proliferation, migration, invasion, and other malignant behaviors, leading to malignant progression of the tumor [16, 20]. Meanwhile, survival analysis was conducted and it was uncovered that PAR2 promoter hypomethylation indicated poor prognosis of LUAD patients (Figure 1(e)), and patients with PAR2 promoter hypomethylation as well as high mRNA expression had significantly shorter survival time than those with hypermethylation as well as low expression (Figure 1(f)). In addition, combined with corresponding clinical information, the promoter methylation level of PAR2 was altered in different tumor stages as well as T stages (Figure 1(g)). Furthermore, three DNA methylated sites (cg00499700, cg14619949, and cg18586277) were noted, and their methylation levels were verified to be reversely related to the PAR2 gene expression level (Figure 1(h)). In view of the abovementioned, we speculated that PAR2 promoter methylation level is relevant to malignant progression of LUAD, while its level shows a relationship with PAR2 gene expression level.

### 3.2. PAR2 highly expressed and hypomethylated in LUAD cells

**Figure 2:**
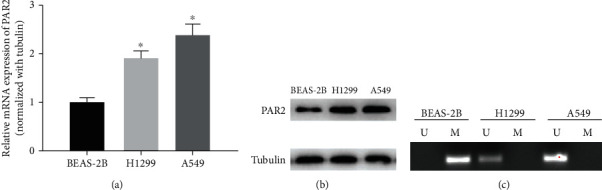
PAR2 has increased mRNA expression and decreased promoter methylation in LUAD cells. (a) qRT-PCR and (b) Western blot results show the mRNA and protein expression of PAR2 at the cellular level. (c) MSP was carried out to determine the methylation of PAR2 promoter in each cell line (U: unmethylated; M: methylated). (^∗^*p* < 0.05).

To validate the promoter methylation and expression levels of PAR2 in LUAD in the cellular level, qRT-PCR and Western blot were run first. It turned out that in comparison with the normal BEAS-2B cell line, H1299 and A549 cell lines had significantly elevated mRNA and protein expression of PAR2 (Figures 2(a) and 2(b)). Additionally, MSP was performed, the results of which revealed that methylation of PAR2 promoter was relatively high in the BEAS-2B cell line, while that in both LUAD cell lines presented as hypomethylation (Figure 2(c)). Considering the above findings, we believed that PAR2 expression in LUAD cells was upregulated while its promoter was hypomethylated.

### 3.3. PAR2 Promoter Hypomethylation Promotes PAR2 Expression in LUAD Cells

**Figure 3:**
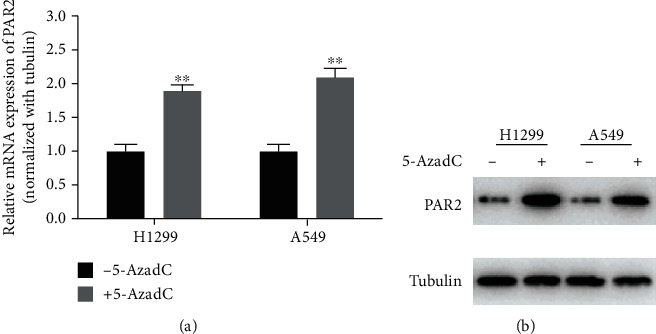
PAR2 promoter hypomethylation promotes PAR2 expression in LUAD cells. (a) qRT-PCR and (b) Western blot results present PAR2 mRNA and protein levels in 5-AzadC-treated cells. (^∗∗^*p* < 0.01).

For the purpose of clarifying the inhibitory effect of the methylation level of PAR2 promoter on PAR2 mRNA expression, DNA methyltransferase inhibitor 5-AzadC was used to treat H1299 and A549 cells. As analyzed, PAR2 mRNA and protein expression were considerably increased in 5-AzadC-treated cells (Figure 3, *p* < 0.01). This finding elucidated that PAR2 expression in LUAD cells could be promoted when promoter methylation was inhibited.

### 3.4. PAR2 Promoter Hypomethylation or mRNA Overexpression Potentiates LUAD Cell Proliferation, Migration, and Invasion

**Figure 4:**
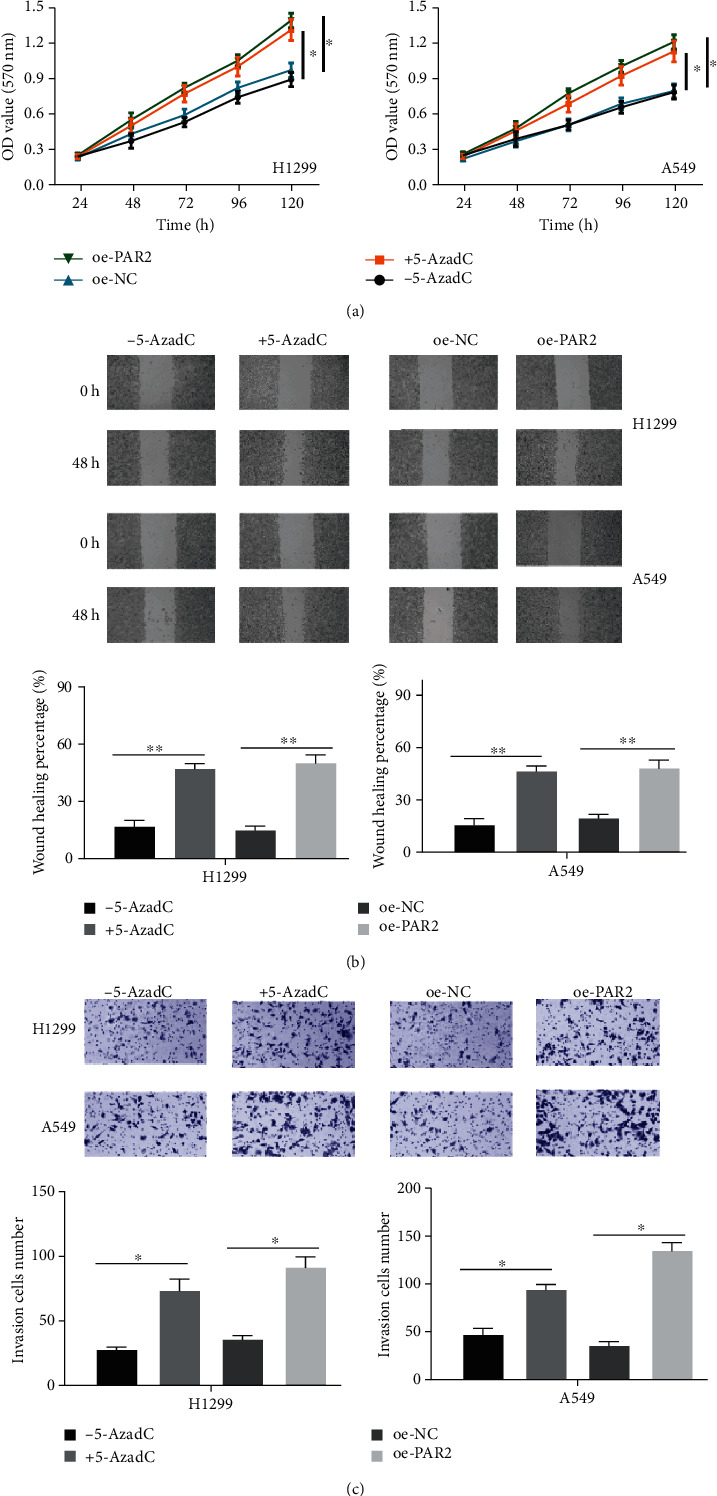
PAR2 promoter hypomethylation or PAR2 mRNA overexpression potentiates LUAD cell malignant progression. 5-AzadC, oe-PAR2, and their controls were used to treat H1299 and A549 cells. Cells were harvested for (a) MTT, (b) wound healing assay, and (c, 100x) Transwell assay to assess cell proliferative, migratory, and invasive abilities. (^∗^*p* < 0.05, ^∗∗^*p* < 0.01).

In this part, associations between promoter methylation/mRNA expression of PAR2 and LUAD cell progression were identified. MTT assay revealed that the proliferative viability of cells treated with 5-AzadC or oe-PAR2 was significantly enhanced (Figure 4(a), *p* < 0.05). In addition, as uncovered by wound healing assay and Transwell assay plotted in Figures 4(b) and 4(c), cell migratory and invasive abilities were both greatly increased in 5-AzadC-treated cells or cells with PAR2 overexpression. Taken together, it could be seen that PAR2 promoter hypomethylation or mRNA overexpression was beneficial for LUAD cell proliferation, migration, and invasion.

## 4. Discussion

In recent years, since the late diagnosis and unfavorable prognosis of LUAD are becoming more common, biomarkers of relatively high effectiveness are in need for the prediction of LUAD outcomes. Cancer-associated mRNAs, as increasing research indicated, are potential markers available in cancer diagnosis and survival prediction, showing vital treatment value [21]. Nevertheless, methylation-driven mRNAs have been rarely touched for their underlying role and molecular mechanism in LUAD so far.

PAR2 is a G protein-coupled receptor activated by serine proteases [22]. As reported, PAR2 shows high expression in diverse cancers including LUAD, and it is relevant to cancer progression and metastasis [23–25]. Arisawa and other researchers [20] found that in gastric cancer tissue, PAR2 promoter is in a low methylation level, while increased promoter methylation will contribute to increased chronic inflammation and suppressed cancer progression. DNA methylation, a kind of epigenetic modification [26], functions in cancer by inhibiting tumor-suppressor genes and/or promoting oncogenes, relevant to cancer initiation and progression [27]. In terms of the effect of PAR2 promoter methylation level on LUAD progression, there have been no studies devoted to this field.

In the present study, we observed from the bioinformatics perspective that in LUAD, PAR2 promoter methylation level was reversely associated with PAR2 gene expression, and patients having PAR2 promoter hypomethylation as well as high mRNA expression suffered a shorter survival time. Additionally, PAR2 promoter methylation level also varied in different tumor stages and pathologic_T stages. Considering the findings, we reasoned that alteration in PAR2 promoter methylation level may affect the initiation and progression of LUAD.

Many studies, for the past few years, demonstrated the relationship between mRNA promoter methylation and cancer development. For instance, Su et al. [28] identified CLEC14A, which is with relatively high promoter methylation level and decreased gene expression level, in LUAD tissue and found that elevating CLEC14A gene expression or suppressing promoter methylation can hinder LUAD cell DNA replication, resulting in cell cycle arrest in G0/G1 phase, inducing cell apoptosis, and suppressing cell migration and invasion. Lin et al. [29] focused on RILP and found that hypermethylation of RILP will cause RILP gene silence, while RILP gene downregulation can help proliferation, migration, and invasion of lung cancer cells. Wang et al. [30] applied methyltransferase inhibitor 5-AzadC to treat LUAD cells to inhibit promoter methylation of target gene SAMHD1, showing that SAMHD1 promoter hypomethylation led to increased SAMHD1 gene expression while suppressed cancer cell proliferation. Here, we also applied 5-AzadC to treat LUAD cells. As analyzed, PAR2 gene expression was significantly elevated in the presence of 5-AzadC, and the proliferation, migration, and invasion of cancer cells were in turn enhanced. These results further proved that in LUAD, PAR2 promoter hypomethylation can increase PAR2 gene expression, thus to facilitate cancer cell proliferation, migration, and invasion.

Generally speaking, the present study identified the promoter hypomethylation of PAR2 in LUAD tissue and its negative relationship with PAR2 gene expression. In addition, this study also proved that inhibiting PAR2 promoter methylation can enhance PAR2 gene expression in LUAD cells and, in turn, potentiate cancer cell proliferation, migration, and invasion. Our research findings may extend our knowledge on the role of PAR2 promoter methylation in LUAD. However, there are still some limitations that need to be improved in the future. For example, we predicted three methylated sites that may affect PAR2 promoter methylation but have not did further verification. In our future studies, we will design experiments to prove this result and identify the specific site responsible for PAR2 aberrant promoter methylation, aiming to enhance the theoretical basis for PAR2 as a potential therapeutic target for LUAD.

## Data Availability

The data used to support the findings of this study are included within the article. The data and materials in the current study are available from the corresponding author on reasonable request.

## Supplementary Materials

Supplementary Materials Supplementary Table 1: primers used in MSP and qRT-PCR. Supplementary Table 2: antibodies used in Western blot.
