# Supplementary material for: PAR2 Promoter Hypomethylation Regulates PAR2 Gene Expression and Promotes Lung Adenocarcinoma Cell Progression
Source: Comput Math Methods Med. 2021 Apr 15;2021:5542485. doi: 10.1155/2021/5542485 (PMC8081642; doi:10.1155/2021/5542485)
Supplement: Supplementary Materials — Supplementary Table 1: primers used in MSP and qRT-PCR. Supplementary Table 2: antibodies used in Western blot. [file 5542485.f1.pdf]

Supplementary Table 1 Primer sequences in MSP and qRT-PCR

**methylation**	**sense sequence**	**anti-sense sequence**
PAR2 (methylated)	5'-TAGGAAAGGTAAGGGAGATTGACGA-3'	5'-AAACTCGCGCCAAATCCCTTAAA-3'
PAR2 (unmethylated)	5'-TAGGAAAGGTAAGGGAGATTG ATGATT-3'	5'-CAAACTCACACCAAATCCCTTAAACT-3'
**qPCR**	**sense sequence**	**anti-sense sequence**
PAR2	5'-GATGGCACATCCCACGTCACT-3'	5'-TTGGCAAACCCACCACAAACAC-3'
Tubulin	5'-TTGACTGGGAGTGAGATTGGA-3'	5'-CAAAATAGCGTTCAGCCACA-3'

Supplementary Table 2 Antibodies used in Western blot

**Antibody**	**WB**	**Specificity**	**Company**
Tubulin	1:1000	Rabbit monoclonal	Abcam, China
PAR2	1:1000	Rabbit monoclonal	Abcam, China
IgG	/	Goat anti Rabbit	Abcam, China
